# Application of MobileNetV2 to waste classification

**DOI:** 10.1371/journal.pone.0282336

**Published:** 2023-03-16

**Authors:** Liying Yong, Le Ma, Dandan Sun, Liping Du

**Affiliations:** School of Mechatronic Engineering, Harbin Vocational & Technical College, Harbin, Heilongjiang, People’s Republic of China; Menoufia University, EGYPT

## Abstract

Today, the topic of waste separation has been raised for a long time, and some waste separation devices have been installed in large communities. However, the vast majority of domestic waste is still not properly sorted and put out, and the disposal of domestic waste still relies mostly on manual classification. The research in this paper applies deep learning to this persistent problem, which has important significance and impact. The domestic waste is classified into four categories: recyclable waste, kitchen waste, hazardous waste and other waste. The garbage classification model trained based on MobileNetV2 deep neural network can classify domestic garbage quickly and accurately, which can save a lot of labor, material and time costs. The absolute accuracy of the trained network model is 82.92%. In comparison with CNN network model, the classification accuracy of MobileNetV2 model is 15.42% higher than that of CNN model. In addition, the trained model is light enough to be better applied to mobile.

## Introduction

### Background and significance of the subject

Waste separation is the key to solving many environmental problems, so residents are encouraged to separate their household waste, but there are so many different types of household waste that many people are unable to properly separate their waste. Initially, people worked to standardize waste separation standards, considering the ease of disposal and carbon footprint [1]. Later, smart bins and kiosks were invented for automatic waste classification [2], but problems such as their poor popularity were also evident. In recent years, the rise of image classification tasks has provided a new direction to solve the garbage classification problem [3]. By using convolutional neural networks to train classification models for a large number of images of household garbage, the obtained models can quickly classify unseen garbage. In order to improve the classification accuracy, the number of network layers has been increased, and the consequent problem is that the size of deep convolutional neural networks is too large to limit the practical applications [4].

In contrast, the emergence of lightweight convolutional neural networks targeted to solve the problems of model size and training speed. The solution of this problem greatly extends the application scope of deep neural network models. The classification query of household garbage fits the application scenario of lightweight convolutional neural network. The application of deep convolutional neural networks to mobile is the significance of this study.

### Innovation points and theoretical contribution

Deep-level neural networks need to be more widely used in mobile instead of just staying in the experimental stage. In this paper, a WeChat applet is developed based on the MobileNetV2 network so that users can get rid of the constraint that large-scale deep neural networks cannot be applied on mobile and can query the type of garbage by uploading the image of household garbage only through mobile WeChat. The MobileNetV2 model used in the study has a 15.42% higher classification accuracy compared with the CNN model in the comparison experiment.

The main contribution and novelty of this paper is the training of lightweight network models and the interconnection with the WeChat applet. Most of the applications about deep-level neural networks are complicated due to the efforts to improve the performance of neural networks. This paper provides a new idea for the application of deep-level neural networks by ensuring that the accuracy of classifying and identifying garbage is in line with the actual situation, and by making the development of a more lightweight network and a more concise front-end applet to make it more convenient for users to achieve the purpose of fast garbage classification. Compared with the current focus on improving the performance of the network, we focus more on how to make the current network get better application. We apply the cutting-edge algorithms of deep learning to the WeChat applets that people often use to help people achieve the correct garbage sorting work, and research to focus on technical hotspots and serve the public from practical problems.

### The arrangement of the full paper

Section1 is an overall introduction of the paper, which explains the Background and significance of the subject, innovation points, theoretical contributions, and the arrangement of the full paper. Section2 of this paper will briefly introduce images classification techniques based on deep learning and the current mainstream applications of deep learning. Section3 will detail this project, from the acquisition and processing of the data set to the training of the model and the development of the WeChat applet. The evaluation results of the model and the effectiveness of the applet for garbage classification are also illustrated. Section4 concludes the paper by explaining the conclusions and shortcomings of this experiment.

## Related work

### Image classification method

Image classification technology uses computers to simulate humans to classify images according to specific rules. It has a wide range of applications in many fields such as medical [5], agricultural, industrial, and service industries. The study of image classification techniques includes feature extraction of ideas and classification algorithms for images.

The convolutional neuralNET(network) structure is a well-known basic architecture for deep learning in image processing. Since this NET structure requires fewer training parameters and satisfies a significant interaction of neighboring information when extracting information from an image, different features can be automatically extracted during the processing of the picture. Currently, the classical, familiar convolutional neural NETs used for image feature extraction: are the LeNet [6] NET, the AlexNet [7] NET, the VGGNet [8] NET, and the ResNet [9] NET. In visual analytics, CNNs have an essential position as the basic framework, requiring less algorithmic preprocessing and easy transfer learning, which gives them a place in both image and video recognition [10].

Based on the exploration of NET depth from VGG to ResNet, Densnet [11] explicitly proposed a new deep neural NET architecture to improve the gradient vanishing problem, i.e., connecting all the NET layers while ensuring maximum information transfer among the layers in the NET. However, while different NET architectures tend to explore the NET at a deeper level, there is another group of studies that target different aspects to achieve optimization of the NET across the board. In terms of modularity, GoogLeNet [12], Inceptionv3 [13], Inception-ResNet [14], ResNeXt [15], Xception [16]; in terms of attention, SENet [17], scSE [18], CBAM [19]; in terms of automation, NASNet [20], EfficientNet [21]. The performance of the NET is optimized to a certain after a certain level of performance optimization of the NET, scientific research starts to focus on the computation time of the NET. At this stage, some scientific researches focus on reducing the computational cost of the actual operation of the NET, so there are efficient NETs such as SqueezeNet [22], MobileNet [23], ShuffleNet [24], etc. NETsfor different concerns are available for selection for different application scenarios, and convolutional neural NETs are rapidly developing in the field of image classification.

### Applications of deep learning

Deep learning is an important branch of artificial neural NETs, and with the rapid development of deep learning, its applications have spread to various industries, such as language assistants, autonomous driving, face recognition, etc. In this paper, we mainly make a review of the application areas of computer vision.

In image classification, the classical deep learning NET models are VGG series, Inception series, and ResNet series. The principle is that the input is image data, and the output is the probability distribution of the current sample belonging to each category. The category with the highest probability is usually selected as the prediction class of the sample. The techniques for image classification have been discussed in detail in the previous subsection. Deep learning is also widely used in target detection, and many researchers are keen to continuously improve the target detection performance of the NET, which makes deep learning develop very rapidly in this direction, and the common classical NET models are RCNN series, YOLO series, etc.

The biggest difference between target detection and image recognition is that not only classifying the image, but also detecting the location of the object. Bounding box is usually used to represent and classify the objects in the bounding box. In addition, FCN and U-net models in the direction of semantic segmentation, C3D and TSN models in the direction of video understanding, and VAE series and GAN series models in the direction of image generation are also familiar deep learning applications. Among them, semantic segmentation is understood as classifying each pixel point, and it is an algorithm to segment the picture and recognize the content in the picture. Video understanding usually performs tasks such as classification of videos, behavior detection, and video extraction of topics. Image generation applications are mainly used to learn the distribution of real images, and sample and obtain the generated images with high fidelity from the learned distribution. In this paper, we combine the application of deep learning in image classification with the current social hot topic of spam classification, and many other researchers work on combining the application of deep learning with social hot topics, for instance, the detection of coronavirus [25], the detection of fake news on the Internet [26], the detection of social accounts [27], haze visibility enhancement [28], etc.

The existing research results focus on theory and on the continuous improvement of the performance of network models. Only a few studies consider the environments in which these networks can be applied. In this paper, we apply deep neural networks to waste sorting efforts, combining theory with practice and making the evolving technology available to serve people.

## Method and results

### Dataset generating and processing method

The research in this paper selects images that are more suitable for practical application conditions as the dataset since it is more oriented to the application of the algorithm. The dataset used in this project is to crawl publicly available images on the Internet using python crawler technology, mainly using requests and BeautifulSoup functions to crawl web information, and then manually checking and organizing it after crawling. The dataset complies with the terms and conditions of the website used. The link to the dataset is publicly available in the Data availability section.

For deep learning, building a satisfactory model selection dataset is crucial. The way of classifying garbage varies from region to region. This paper’s most common classification method, i.e., domestic refuse, is divided into four major categories: recyclable waste, kitchen waste, hazardous waste and other waste. Since there are many types of daily household waste, it is essential to include as many types as possible when selecting the dataset. After determining the search target, many open-source dataset websites were searched, and some open-source datasets were downloaded. However, the quality of the images varied and contained many bad photos, so the subsequent work was to organize these datasets with the data crawled by myself and save them all in jpg format. The sorted dataset contains more than 80,000 images of household waste.

Due to the large number of images in the dataset, this paper divides the pictures into four major categories to improve the model’s accuracy. Also, it separates the whole dataset into 245 smaller categories, i.e., major category labels and minor category labels. The household waste in the dataset is named as “major category label_ minor category label”. The primary category labels are recyclable, kitchen, hazardous, and other wastes, and the minor category labels are the actual names of the waste. For example, “Kitchen waste_ apple”, “Recyclable waste_ paper box”, “Hazardous waste_ battery”, “Other waste_ pen”, etc. This is a good division for the training and testing of the model. And then, the Python program was written to divide the dataset into a training set, validation set, and testing set in the ratio of 8:1:1.

**Fig 1:**
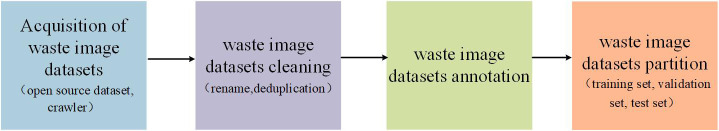
Data set processing flow. The flowchart shows, from left to right, the acquisition, cleaning, annotation and partitioning of the waste image datasets. The images are original and copyright free.

The critical step of dataset processing is to divide the images into a training set, validation set, and test set according to 8:1:1. First, a new dataset location is generated under the directory of the original dataset, which contains three parts: “train”, “val”, and “test”. To facilitate the subsequent evaluation of the model, the three parts are divided to save the training set images, validation set images, and test set images. Then the same directory structure as the original dataset is created in the target folder, i.e., the folder format is “primary title_ secondary title”. Finally, we start to divide the dataset by disrupting the order of the dataset, sampling it in layers, and then traversing it by the size of the training set, validation set, and test set, copying the data images from the original folder and pasting them into the newly generated corresponding folder. The processing flow of the dataset is shown in Fig 1.

### Garbage classification model

**Fig 2:**
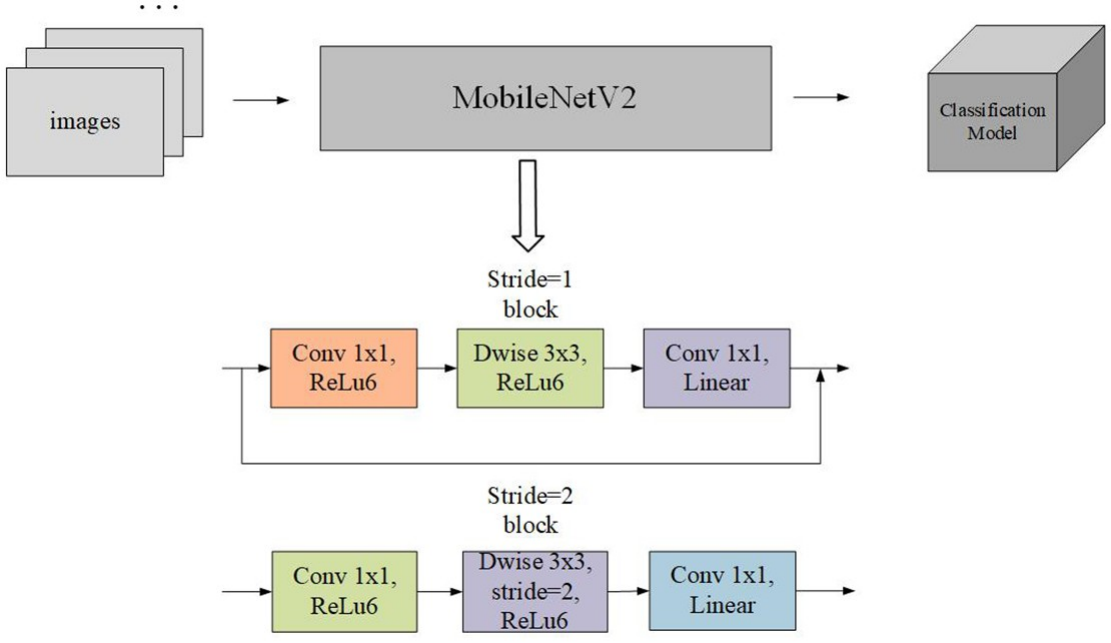
MobileNetV2 network structure. The figure shows that unlike the conventional ResNet network, this network has shortcut connections only when stride = 1 and the input and output feature matrices have the same shape. The images are original and copyright free.

In this paper, based on the consideration of the mobile application, the lightweight MobileNetV2 [29] network model is chosen for migration learning. The structure of the classical lightweight MobileNetV2 network model is shown in Fig 2. Depth-level separable convolution is the basic structure of the MobileNets network, but in real applications, only deep separable convolution is not enough; the mobilenetv1 [30] network also adds a batch norm module and uses the relu activation function. The Google team proposed a MobileNetV2 [31] network in 2018 compared with The highlight of the MobileNetV1 network is the use of depth-level separable convolution and the addition of two hyperparameters to slim down the model. Still, its network structure is similar to that of the VGG network, without a connection similar to the shortcut in the ResNet network. Some users reported that the DW convolution in the MobileNetV1 network is quickly scrapped during the training process, and the network effect is unsatisfactory. MobileNetV2 network is based on MobileNetV1, and the structure is adjusted by introducing Inverted Residuals and Linear Bottlenecks.

**Fig 3:**
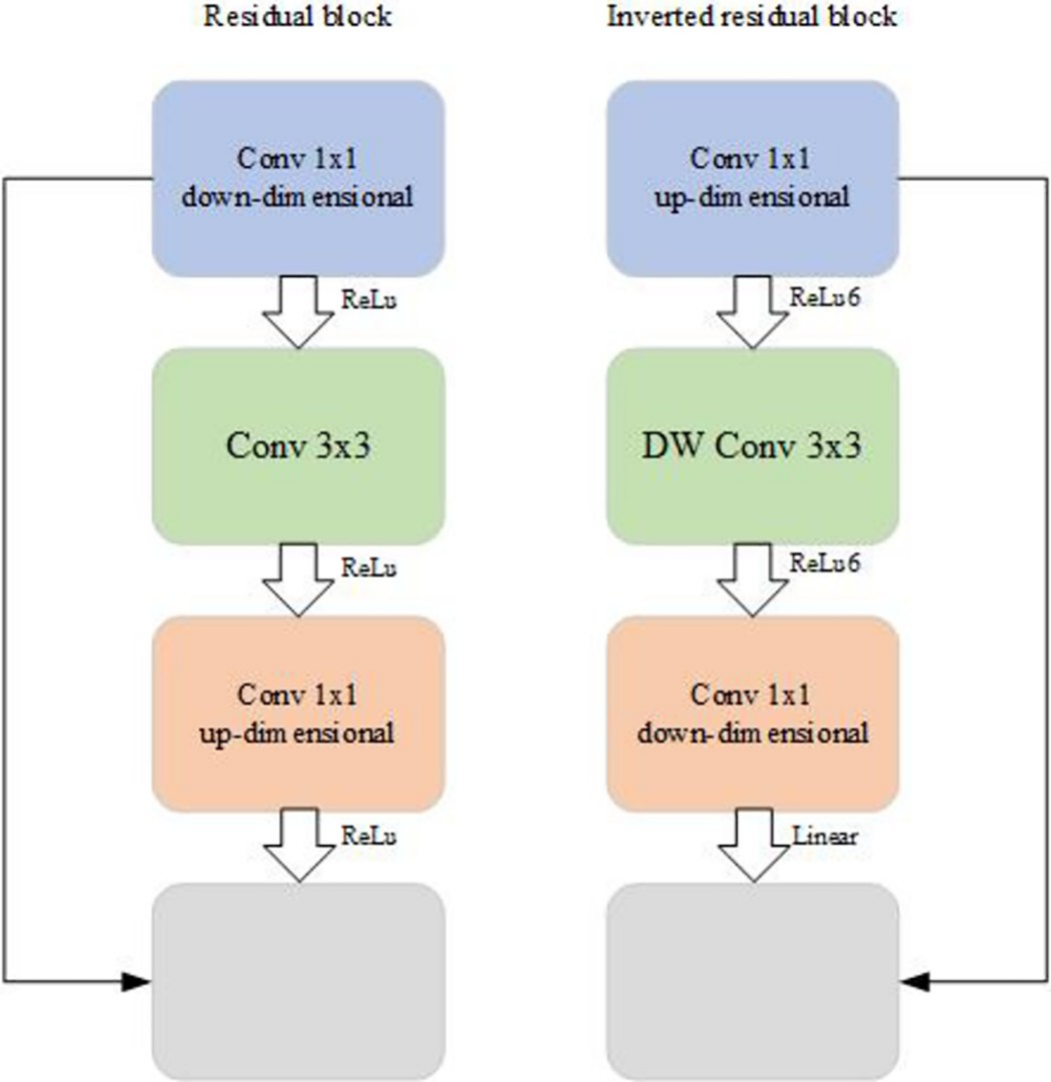
Residual structure and inverse residual structure. In the structure of Residuals proposed by ResNet, 1 × 1 convolution is used to achieve dimensionality reduction, then 3 × 3 convolution, and finally 1 × 1 convolution to achieve dimensionality increase, resulting in a structure with two large ends and a small middle. The Inverted Residuals structure reverses the order of dimensionality reduction and dimensionality increase, and replaces the 3 × 3 convolution with 3 × 3 DW convolution to obtain a structure with two small ends and a large middle. The images are original and copyright free.

The Inverted Residuals structure highlights the entire MobileNetV2 design, which has the opposite system compared to the Residuals structure [32]. In the Inverted Residuals structure, it starts from Convolutional Downscaling to DW Convolution and finally Convolutional Upscaling, while in the Inverted Residuals structure, it begins from Convolutional Upscaling to DW Convolution and finally Convolutional Downscaling. The structure of Residuals and Inverted Residuals is shown in Fig 3. Since the core of the MobileNetV1 network is the depth-level separable convolution, this convolution method can significantly reduce the computation and the number of parameters. The structure will dramatically improve the performance of MobileNetV2. In addition, the inverse residual design uses the ReLU6 activation function, but the final convolution layer uses the linear activation function. The last convolution compresses the feature map before the activation function, and the ReLU activation function outputs 0 for negative input values, which can cause information loss. Using the Linear activation function can reduce the lost information.

The MobileNetV2 network uses the ReLU6 activation function with the Linear activation function. The Rectified Linear Unit (ReLU) is the most frequently used activation function today. It has the advantage of faster practice rate and freedom from gradient explosion and gradient disappearance compared to other common activation functions. Based on the ReLU function, the ReLU6 function has emerged to provide a better fulcrum for applying the model to mobile. In this paper, the study hopes that the classification model is applied to mobile devices, and it is very suitable to use the ReLU6 function as the activation function. the ReLU6 function formula is shown in Eq 1.

$$\begin{array}{c} ReLU6=\text{min}(6,\text{max}(0,x)) \end{array}$$ (1)

The Linear function is a linear activation function. Since the inverse residual structure of the MobileNetV2 network is a low-dimensional feature at the final output, a linear activation function should be used instead of a nonlinear activation function to prevent destroying too much information. The formula for the Linear function is shown in Eq 2.

$$\begin{array}{c} y=b+\sum_{i}x_{i}w_{i} \end{array}$$ (2)

In this paper, after selecting the MobileNetV2 network, we used the open-source code from the Gitee website to do migration learning on the pre-trained model with the processed dataset. The built MobileNetV2 network was trained using an RTX3060 graphics card with i7–11800H Processor, and the divided dataset was prepared using the MobileNetV2 network. The model’s accuracy was improved with an increasing number of iterations after several adjustments. The model’s accuracy was evaluated comprehensively with the cross-entropy loss function, the number of iterations was 50, and the expected results were obtained from the classification model.

### The development of WeChat applets

WeChat Mini Program [33] is an application that is simple to develop and efficient. When writing the program, WXML is applied to define the content of the program; WCSS [34] is used to set the appearance of the program; JavaScript [35] is applied to obtain data and update the language of the web page in real-time; JSON [36] is used to realize data exchange with the back-end model. In the development process of the applet, the page visualization, operation flow, and data exchange with the applet’s back-end model are the design’s problematic points.

In visual design, an intuitive, simple, and easy-to-operate interface should be designed according to the actual use scenario, and the graphic design directly determines the user experience. After referring to several applet interfaces related to query information and many successful graphic designs through Google search, we have an overall vision for the visual design of the waste sorting applet studied in this paper. A sound applet does not have a complicated and fancy graphic design. Still, it starts from the actual user, from the real application scenario, so that the screen is simple to meet the needs of the audience group. After opening the applet, you can see the eye-catching process tips; the screen is beautiful and easy to operate. The design in this paper starts from the operation interface and logo graphics, designed to meet users’ needs to check the waste category anytime and anywhere and to enhance the user experience as much as possible.

In the process design, the convenience and speed of the process directly affect the user’s experience. One of the advantages of the WeChat applet is that it takes WeChat as the big platform, so there is no need to download or register, which does not require too much cell phone performance and storage space for the users of the WeChat applet and meets the advantages of lightweight use. In the layout and process design, the layout is also smooth and reasonable, and the operation is easy and fast. After entering the app, click into the category, select the image to be identified and upload it. Then, the type of waste will be displayed. There is no redundant operation in the whole process, so it won’t take too much time because of the complicated procedure, which can be used in practice to find out the category of waste instantly when putting out the trash and meet the demand of users to put out the trash after using the app anytime and anywhere.

In terms of interaction design, one of the significant difficulties of the applet design is how to make the applet call the back-end model to recognize the images uploaded by the front-end and how to realize the applet for multiple systems on the mobile end. In this paper, the interaction design uses the Flask [37] framework to connect the front-end to the back-end of the project, which receives the data from the front-end and transmits it to the back-end of the application, which then recognizes it and sends the recognition result back to the front-end interface. In addition, the Flask framework, compared to other application frameworks there, is robust customizability; because it is only a simple kernel, almost all the features require users to use third-party extensions to achieve, which is precisely one of the advantages of the Flask framework, users can design according to their own needs to add the corresponding features so that the Flask framework to achieve the core of simple and feature-rich features. Flask framework is a powerful extension that is also unmatched by other frameworks.

**Fig 4:**
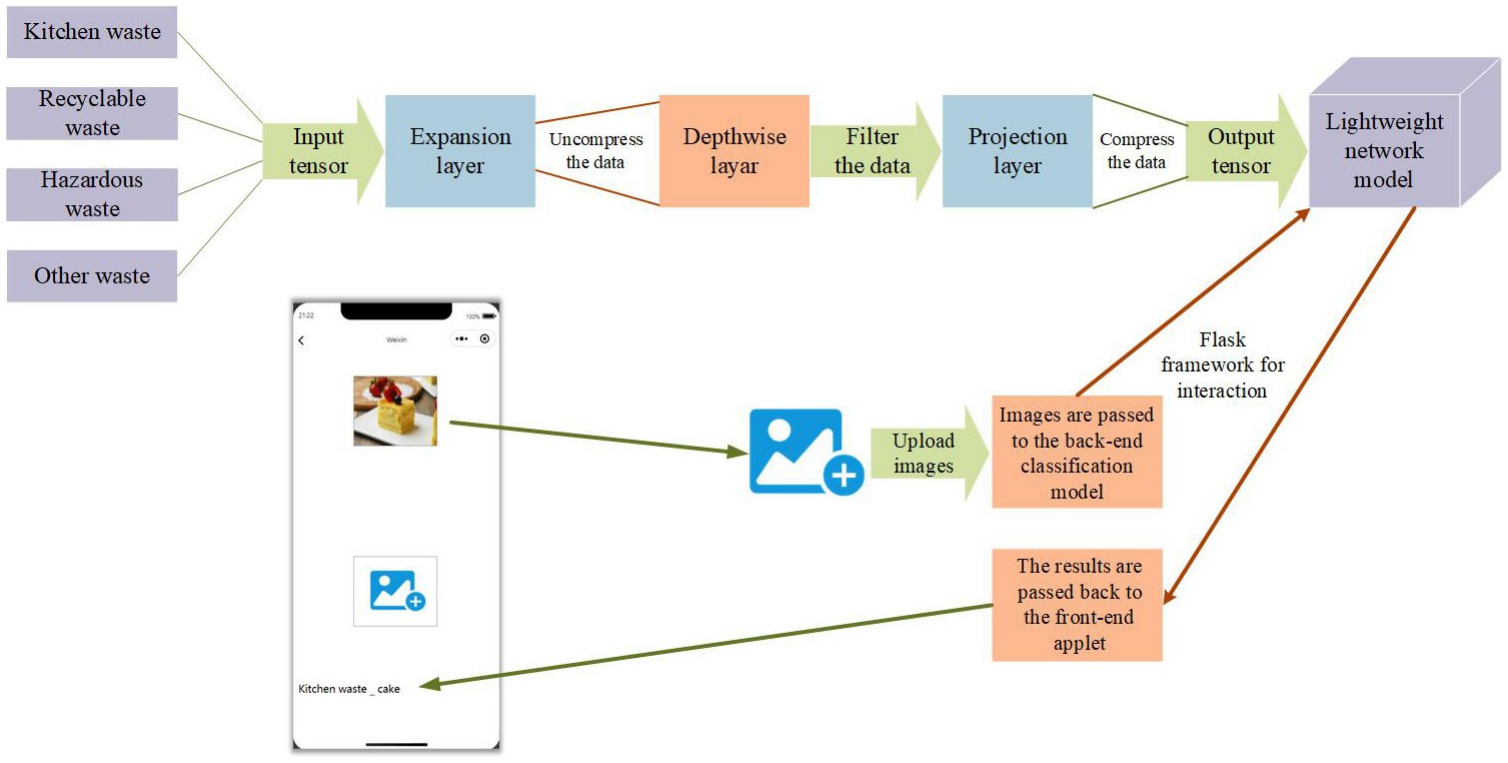
System architecture figure. This architecture flowingly describes the work of this paper. The process of training the model and calling the model by the applet are explained. The images are original and copyright free.

The system architecture figure is shown in Fig 4. In this paper, over 80,000 images of household waste are classified into four categories: food waste, recyclable waste, hazardous waste, and other waste. The data set is pre-processed and put into MobileNetV2 network for migration learning, and the classification model is trained and then the parameters are repeatedly adjusted to obtain the optimal model. In addition, we developed a user-friendly WeChat applet, visual design, flow design and interaction design for the applet, which used the Flask framework for front and back-end connection. When users use the WeChat applet for waste classification recognition, they first upload the image to be recognized, and the system will pass the image to the back-end model for classification recognition, and then pass the result back to the front-end applet after the classification model gets the result, and the applet will display the classification result to the user.

### Waste classification results

**Fig 5:**
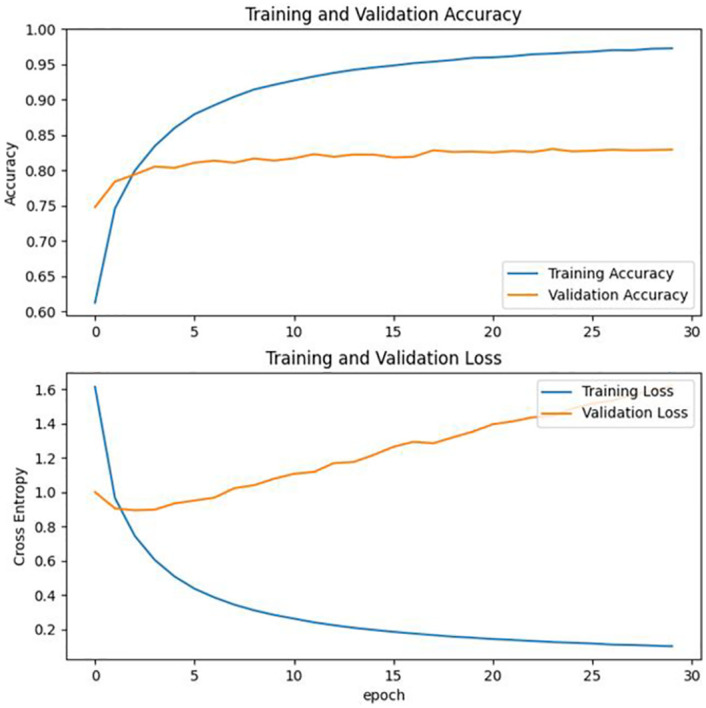
Comparison of cross-entropy loss functions and accuracy. When epoch = 30, the cross-entropy loss function and accuracy of the training set and the validation set is shown. The images are original and copyright free.

In this paper, after obtaining the deep neural network classification model, the accuracy and cross-entropy loss function is selected as evaluation metrics to analyze the model’s performance. The model’s accuracy and loss values on the training set after each iteration are recorded during the training process. The model’s performance on the validation set is also recorded. Finally, the data from the process is organized into a txt file. The data shows that the model’s accuracy on the validation set increases as the number of iterations increases. The total number of training iterations was 50, and the expected results have obtained the performance of the classification model. And then, the experimental data were visualized using the Matplotlib [38] module in Python, and the results obtained are shown in Fig 5.

**Fig 6:**
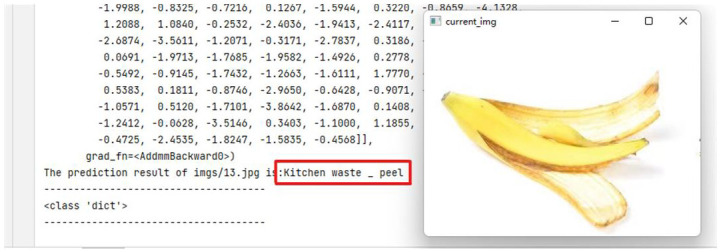
Single picture test result. A generalizability test is performed using the trained classification model, and the classification results are accurate and as expected. The images are original and copyright free.

And then, the model was used to classify the household waste pictures and output the results. The test results obtained are shown in Fig 6, which shows that the classification effect of the model is ideal.

After successfully developing the WeChat applet, you need to configure the back-end model with the front-end applet network API in line, run the main.py file of the back-end will appear the current API address, copy the address to the program code in the WeChat applet, you can use the applet to call the back-end model for waste classification. After successfully debugging the WeChat applet and testing on the applet simulator, it can be seen that the applet can smoothly realize reading the waste pictures that need to be identified, passing them to the back-end classification model, and then getting the results after the model classification to give back to the front-end applet, which displays the classification results.

After the interaction between the back-end model and the front-end applet, the applet is used to test the classification of household waste. The applet can smoothly read the waste pictures that need to be recognized, pass them to the back-end classification model, get the results after the model is classified, and pass them back to the front-end applet, and the applet displays the classification results. From the visual screen, the development of the applet takes into account the beautiful interface and simple operation. It can be used to identify the waste images you want to classify by simply clicking on the uploaded image logo. In the actual use process, users can open the WeChat app on their cell phones at any time when they are unable to classify the waste accurately and put it in, and then take pictures of the trash and upload them. There is no redundancy in the whole process, and the user can quickly locate the uploaded logo on the screen, making classification waste simple and straightforward.

**Fig 7:**
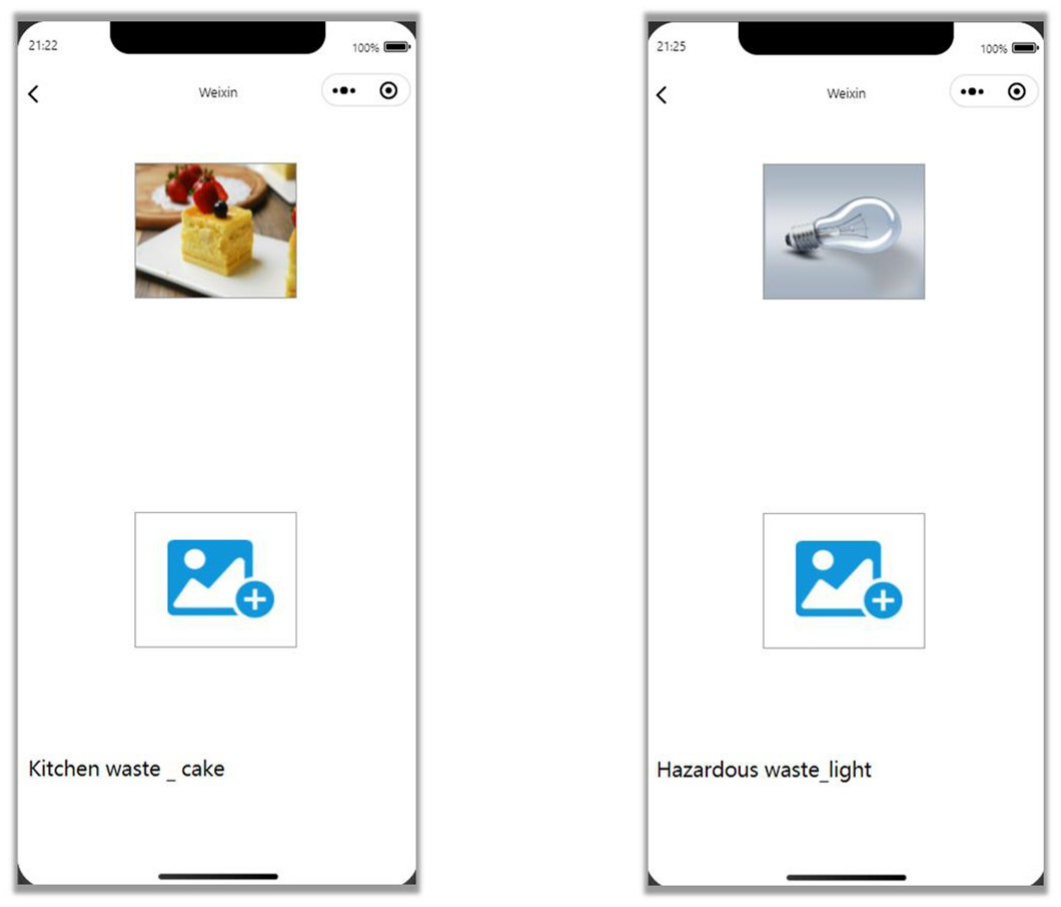
Waste sorting results. The results of the waste classification test using the developed WeChat app indicated that the classification results were accurate and as expected. The images are original and copyright free.

The results of the test experiments on the WeChat applet simulator are shown in Fig 7. In terms of the accuracy of the classification results, the trained model has high classification accuracy, good classification effect, and excellent generalization performance. The time required for the whole classification process is concise, which is attributed to the lightweight of the model and the fact that the model has promising results on the CPU both in terms of network acquisition and its running speed.

**Fig 8:**
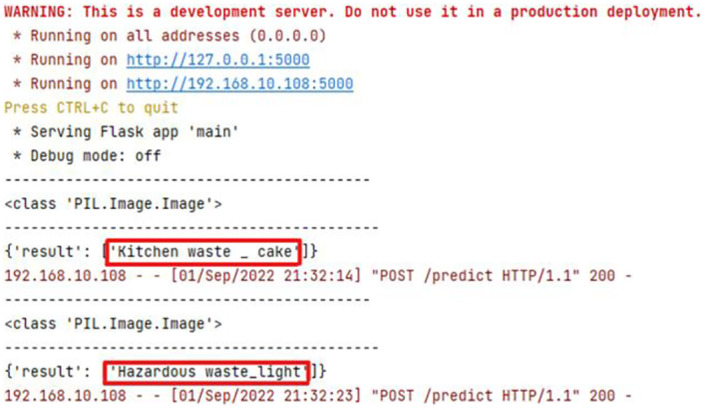
Back-end model classification of front-end image results. The classification results of the back-end model are recorded, when using the WeChat applet for waste classification. The images are original and copyright free.

The results of the back-end model’s classification and recognition of waste while testing experiments on the WeChat applet simulator are shown in Fig 8. The whole process is efficient and effective. So far, the investigation has got the expected result, users can use the applet to realize the instant query of daily household waste, and after the question, they can put out the trash according to the classification result, which meets the expected goal.

**Fig 9:**
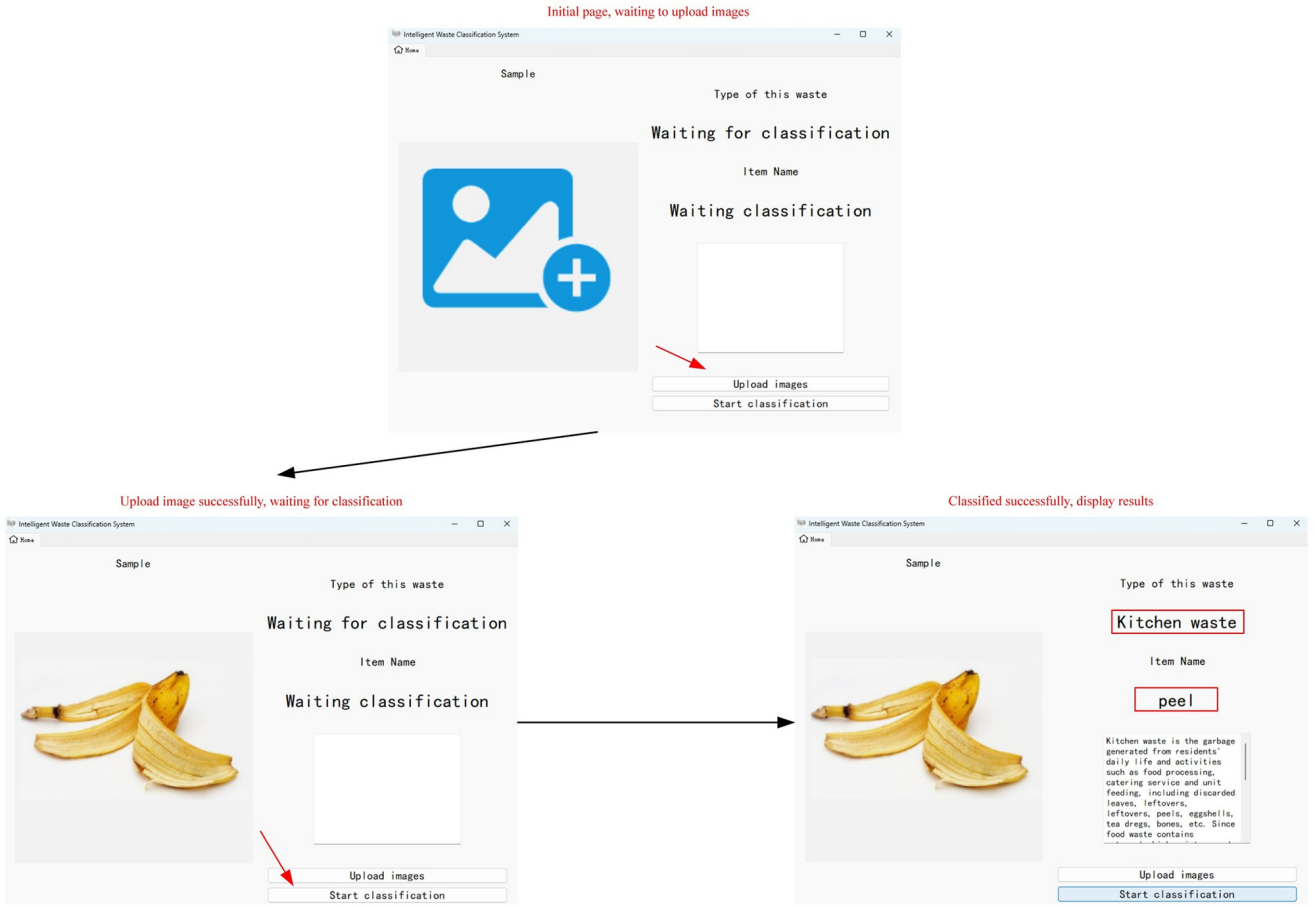
Operation process diagram. The operation flow chart of the web version of the waste classification inquiry window.

In addition to the application on the mobile WeChat applet, the study also designed a visualization window for waste classification query to illustrate that the web model can also be applied on the web side and the application scenario can be further expanded. The visualization window is developed by Pyqt5 library, and the operation steps of the waste classification query are shown in Fig 9.

### Comparison experiment

**Fig 10:**
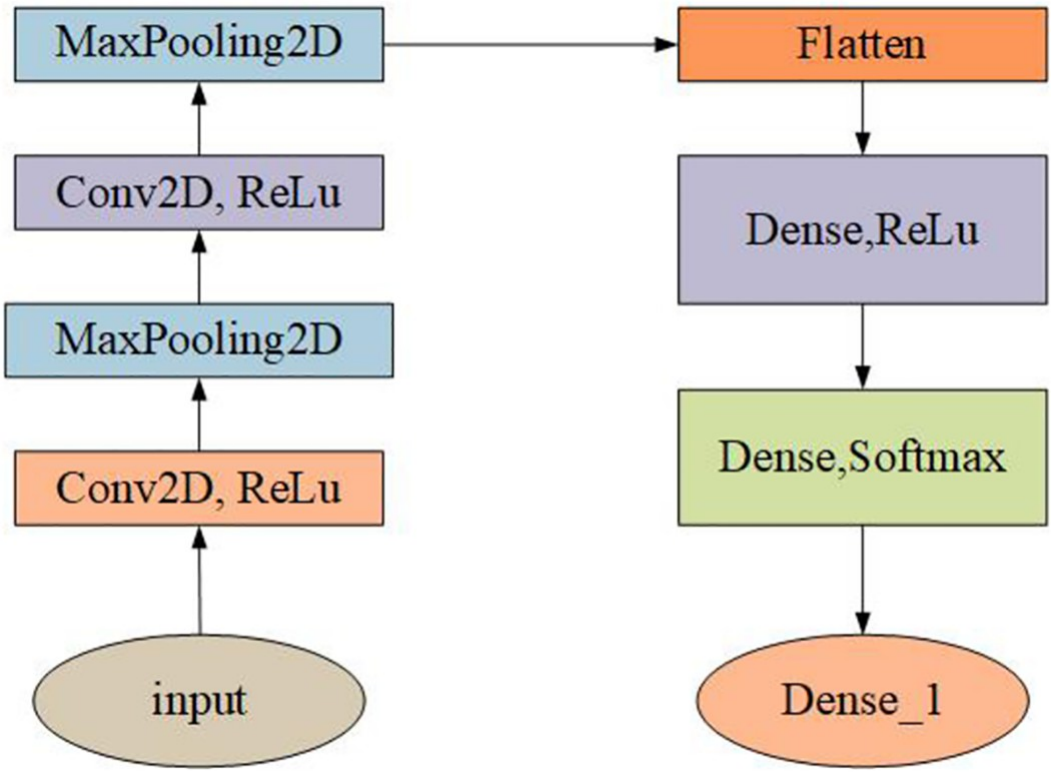
CNN model network structure. This is a classical CNN model consisting of two layers of convolution and two layers of pooling, where features are straightened and fed into two fully connected layers for classification. The images are original and copyright free.

In order to prove that our model can help people to sort waste well, we set up a comparison test. The model used for the comparison is the classical CNN network model, and the model structure diagram of the network is shown in Fig 10.

**Fig 11:**
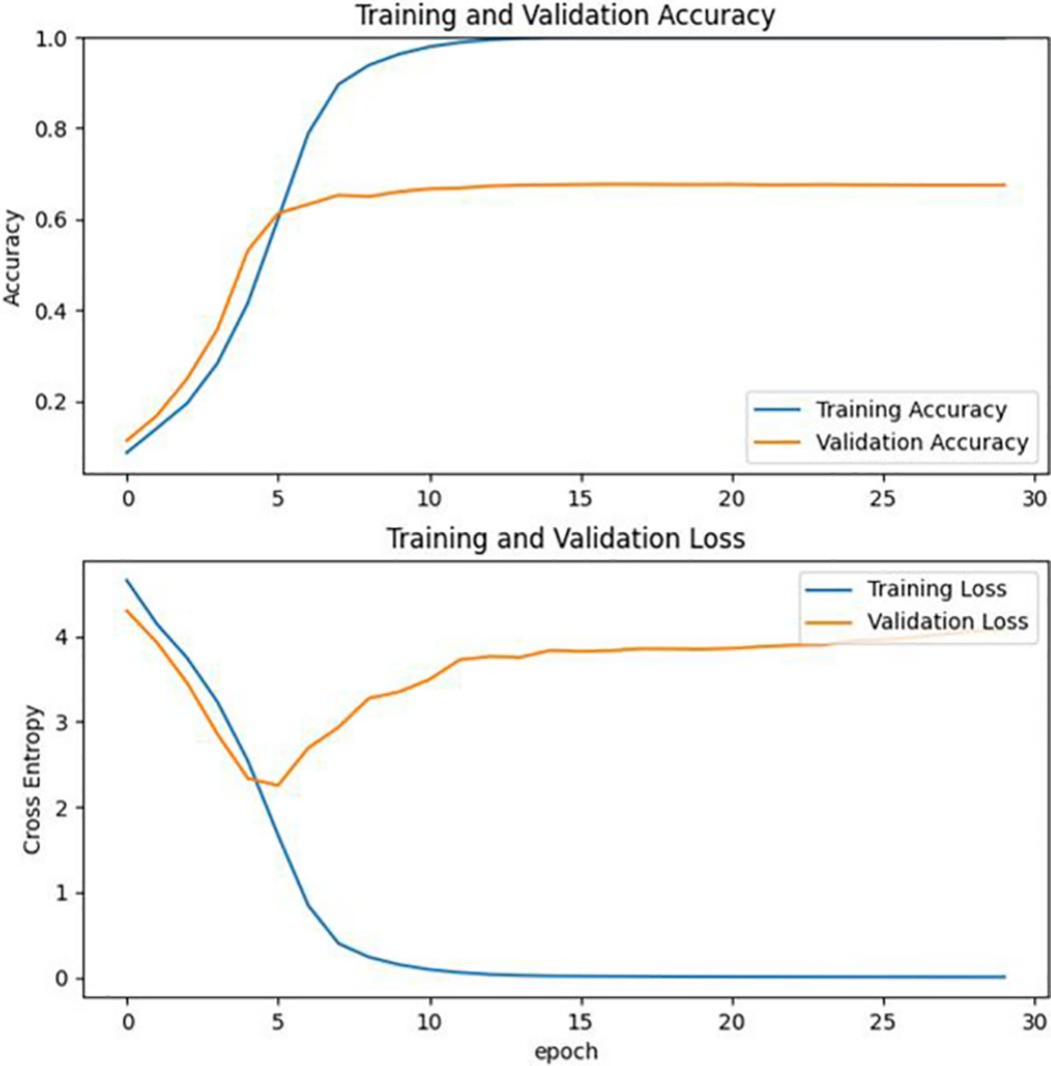
Comparison of cross-entropy loss functions and accuracy. When epoch = 30 for CNN, the cross-entropy loss function and accuracy of the training set and the validation set is shown. The images are original and copyright free.

CNNs are mainly used to identify 2D graphics that are offset, deflated and otherwise distorted from invariance. Because the CNN feature detection layer learns the training data, the use of CNN avoids indicating the svm algorithm and instead learns implicitly through the training data. Secondly, because the neuron weights on the same feature mapping surface are the same, the network is able to process learning in parallel, that is also a major advantage of convolutional and networks over networks connected in the middle of neurons. Convolutional and neural system networks have special advantages at the level of speech recognition techniques and digital image processing because of their special structure of partial weight sharing. It is equipped with similar specific neural system network configurations and the weight sharing reduces the complexity of the network, especially in is due to the fact that images with multi-dimensional keying space vectors can be directly keyed into the network. Since our model is designed to be used on mobile, the CNN model structure we used for comparison is not deep in the hierarchy, since we had to use a model that can be applied in the same environment as the MobileNetV2 model as a comparison. The classical CNN model was used for training using the same dataset with guaranteed model size. Fig 11 shows the training and validation results of the CNN model.

It can be seen that the accuracy of the CNN model is only 67.5%, and the accuracy of MobileNetV2 is higher, about 83%. A comparison table of the performance of the two is shown in Table 1. In addition, MobileNetV2 model is an efficient lightweight network model with good performance under CPU conditions. Therefore, this study chooses to use MobileNetV2 migration learning to train the classification model. In addition to testing the entire dataset, 20% of the dataset was randomly selected for this study and divided into four categories: kitchen waste, recyclable waste, hazardous waste, and other waste. Three metrics, precision, recall and f1 score, were chosen to evaluate the two models.

**Table 1:** Results of the comparison experiment.

Model	accuracy%	cross-entropy loss functions
CNN	67.5	4.075
MobileNetV2	82.92	1.621

Taking kitchen waste as an example, the experiment will be kitchen waste recorded as positive when tested. Precision refers to the proportion of samples predicted to be positive by the model that are also actually positive to the proportion of samples predicted to be positive; recall refers to the proportion of samples predicted to be positive to the proportion of samples that are actual positive; and F1 score is the summed average of precision and recall. The results are presented in Tables 2 and 3 below. The metrics show that the MobileNetV2-based model outperforms the CNN-based model under every category.

**Table 2:** Test metrics for MobileNetV2 model.

Category	precision	recall	f1-score
Kitchen waste	0.98	0.98	0.98
Recyclable waste	0.99	0.98	0.98
Other waste	0.96	0.96	0.96
Hazardous waste	0.92	0.97	0.94

**Table 3:** Test metrics for CNN models.

Category	precision	recall	f1-score
Kitchen waste	0.94	0.97	0.96
Recyclable waste	0.97	0.96	0.97
Other waste	0.89	0.86	0.88
Hazardous waste	0.91	0.88	0.90

## Conclusions

In this paper, we use the lightweight network model MobileNetV2 for migration learning to obtain a deep convolutional neural network model to realize the classification and recognition of household waste and realize the application of the lightweight network model on mobile in combination with the development of WeChat applet. After repeated training, the accuracy rate of the waste classification model can reach 82.92%, which can meet the demand for daily waste classification. In recent years, to solve the problem that deep neural networks cannot be deployed to mobile and embedded devices, many lightweight neural networks have emerged, and this paper also starts with this problem. It combines the hot topic of waste classification as the background. The main impact of this paper is that our research is more about thinking about how to make the current network be used better rather than studying how to continuously improve the network performance. The main contribution of this paper is to combine the lightweight network model with WeChat applets to realize the application of waste classification. The cost-effectiveness of our proposed method is relatively high, and the proposed model can be integrated in APP directly after encapsulation, and the APP program runs in real time, which can achieve good results in the real-time application of waste classification.

This paper’s waste classification model can meet most of the daily waste classification. However, since most of the dataset images available for training are normal form items, low classification accuracy may occur when the household waste to be dropped off is severely deformed. Therefore, it still has some limitations, the system can only classify the common forms of waste, and is not able to accurately classify the waste with large changes in form, especially when the waste is deformed, dirty or broken the system is not able to classify well.

There are three main areas of future research that we are considering. Firstly, in terms of dataset, we should consider expanding the dataset by adding more dataset images that match the shape of household waste at the time of actual placement, so that the model can further learn waste images of various shapes. We will also consider dividing the dataset in different ways and using the k-fold cross-validation method to do more extended studies. Secondly, from the model perspective, the time cost of training the model is an issue that we need to focus on in our future research, and we will consider using the big oh notation method to evaluate the cost-effectiveness of training the model when we need to compare the training time of the model in future updates of the algorithm. Finally, from the perspective of practical application, we consider further development of the WeChat applet, optimizing the operating interface of the applet and extending other convenient services, such as locating the nearest waste drop-off location.

## Supporting information

S1 Data (RAR)
